# Impact of a 10-week multimodal stress management and lifestyle modification program on stress response and immune function in Crohn’s disease: a mixed-methods approach using the Trier Social Stress Test

**DOI:** 10.1016/j.bbih.2025.101006

**Published:** 2025-04-30

**Authors:** Özlem Öznur, Sandra Utz, Christoph Schlee, Jost Langhorst

**Affiliations:** a Department for Integrative Medicine, Medical Faculty, University of Duisburg-Essen, Essen, Germany; b Department of Internal and Integrative Medicine, Sozialstiftung Bamberg, Bamberg, Germany; c Institute for Sociology, University of Bamberg, Bamberg, Germany

**Keywords:** Crohn’s disease, Stress, TSST, Immune function, Randomized controlled trial, Integrative medicine, Mixed-methods

## Abstract

One of the major factors for deterioration and relapse in inflammatory bowel diseases is chronic (psychological) stress. Aim of the present study was to compare the reaction of N = 33 patients with Crohn’s disease that either participated in a multimodal stress management and lifestyle modification program (n = 19) or not (n = 14) to the induction of acute stress after the day-clinic by using the validated instrument of the Trier Social Stress Test (TSST). A mixed-methods approach using self-reported stress perception (questionnaire, qualitative interviews), diary records, and blood samples was applied. Immune and endocrine measures of stress were collected before and repeatedly after stress exposure. Analysis of the blood samples indicated changes in leucocyte and platelet levels only in the intervention group. Differences in the reaction to acute stress might be explained by a significant reduction in perceived (chronic) stress levels in the intervention group compared to baseline (p = .004), whereas there was no change in the control group (p = .472). Diary records (during the day-clinic) showed a notable increase in the number of relaxation techniques (p < .001) and meditative movements (p > .001) performed in the intervention group compared to the control group. In the qualitative interviews (of the intervention group), patients reported a reduction in stress in their daily lives and in acute stressful situations as a result of using the newly learned specific stress management techniques. The observed improvements in stress management (questionnaire, qualitative interviews), indicated by the reduction in perceived stress, and immune function, suggested by the blood sample results, highlight the potential of integrating multimodal stress management and lifestyle changes into the treatment approach for Crohn’s disease patients. Further research is warranted to explore the long-term effects and the multiple mechanisms underlying these observed changes.

## 1 Introduction

Crohn's disease (CD) is a chronic inflammatory bowel disease (IBD) of unknown etiology characterized by periods of activity and remission, often accompanied by an impaired immune response and a reduced quality of life (QoL). Stress has been shown to negatively impact the severity and course of IBD, potentially triggering flares (Langhorst et al., 2005; Torres et al., 2016). Stress occurs when environmental demands exceed an individual's adaptive capacity (Cohen et al., 1997), leading to physical, psychological, or social dysfunctions (European Foundation for the Improvement of L and Working, 2010). It can be classified as distress (unpleasant) or eustress (motivating) (Selye, 1976), and it can manifest itself as acute (short-term) or rather chronic referring to a long-term response to the pressures of life (American Psychological, 2018). Acute stress triggers physical symptoms like rapid heartbeat and sweating, while chronic stress can lead to long-term health issues such as immune suppression, negative impact on memory, and digestive disturbances (Yaribeygi et al., 2017). Chronic stress modifies the sympathetic, neuroendocrine, and immune responses to acute psychological stress in humans (Pike et al., 1997). Psychological stress significantly contributes to the pathophysiology of chronic inflammatory bowel diseases. It is crucial to understand that an imbalance between pro-inflammatory and anti-inflammatory T cell responses plays a key role in CD, since recent studies suggest that psychological stress can influence T cell differentiation and function, thereby affecting this balance (Ge et al., 2022). Stress also modulates the gut-brain axis, for example by increasing intestinal permeability and decreasing immune reactivity, which can worsen IBD (Brzozowski et al., 2016; Labanski et al., 2020). In contrast to short-term stress, high long-term stress triples the risk of exacerbation in ulcerative colitis (Levenstein et al., 2000). The optimal methods for managing stress remain uncertain (Black and Slavich, 2016), but therapy approaches for stress reduction play an important role in non-pharmacological treatment approaches (Labanski et al., 2020). Managing stress is crucial for IBD patients, as about 91 % of them report that stress significantly influences their disease progression (Luo et al., 2018), and 85 % say that effectively managing stress improves their condition (Langhorst et al., 2005). High levels of perceived stress, which focuses on patients‘ subjective perception of feelings and emotional responses (Cramer et al., 2017), is closely linked to diminished QoL in IBD patients (Luo et al., 2018).

Mind-body medicine (MBM) focuses on stress reduction and lifestyle changes. It is shown that mindfulness and stress regulation techniques like yoga, qigong and tai chi have diminished perceived stress in persons with IBD (Cramer et al., 2017; Koch et al., 2020), thereby improving their overall quality of life. Audio relaxation-training sessions were also able to have an effect on stress levels and achieve an improvement in mood and quality of life (Mizrahi et al., 2012). Langhorst et al. (2007a) had shown, that the use of relaxation techniques was a significant predictor of improvement in the psychological sum score after three months of therapy, measured by the SF-36 quality of life questionnaire. Lifestyle modification programs (LSM) include different aspects of mind-body medicine and intend to improve patients’ health and QoL by teaching e.g. coping strategies for stress (Elsenbruch et al., 2005; Langhorst et al., 2007b, 2020). Coping is considered effective when it reduces or eliminates distress. Even if the stressor, like an illness, is still there, the person feels less upset emotionally, socially, or physically. Therefore, multi-modal non-pharmacological approaches with focus on lifestyle modifications seem to be an effective tool in prevention and therapy of IBD (Torres et al., 2016; Labanski et al., 2020; Black and Slavich, 2016; Koch et al., 2021). According to patient reports (interviews), they can be helpful in reducing psychological stress and physical symptoms, and as a consequence improving patients’ overall well-being and QoL (Schlee et al., 2022). The focus on mindfulness and self-care could be one key factor.

Based on these findings, the primary objective of the randomized controlled trial (RCT) by Bauer et al., 2022, 2024, of which this sub-study was a part, was to investigate whether our multi-modal comprehensive lifestyle-modification day-clinic program could improve the quality of life and reduce disease activity of patients with CD as shown in previous studies with IBD. After 10-weeks of the program, patients showed improvements in health-related quality of life (Kirschbaum et al., 1993; Schlee et al., 2024) and in relevant fecal markers (Bauer et al., 2024). Based on these promising initial results, this sub-study aims to clarify the role of stress management in this context.

Can such a program including teaching them adequate stress coping strategies also significantly change the way CD patients handle acute stress and perceive their chronic stress? We hypothesized that the difference in amount of activities learned during the day-clinic might contribute to a better acute stress management in the intervention compared to the control group. We also hypothesized that patients’ own perception of stress could be changed for the better, like already shown for ulcerative colitis (Koch et al., 2021). After the day-clinic intervention (at week 12), patients participated in the *Trier Social Stress Test* (TSST) (Kirschbaum et al., 1993), which is a standard procedure for inducing acute psychosocial stress under laboratory conditions (Allen et al., 2017). The aim was to analyze the effects of the TSST especially regarding perceived stress and physiological stress, using blood parameters to compare the intervention and control group. We hypothesized that patients of the intervention group would overall report less perceived stress as a result of the 10-weeks program, and that they can apply the acquired stress management strategies during TSST. This might be reflected in immune cell counts and cortisol levels, indicating a balanced regulation of psychological stress on both.

## 2 Material and methods

### 2.1 Study design and day-clinic program

The present study presents results of stress-related outcomes of an RCT (Bauer et al., 2022, 2024). A trial with parallel groups randomized allocation of patients with CD in a 1:1 ratio to the intervention (day-clinic program) or control group and two follow-up assessments at 3 and 9 months after the baseline assessment was conducted between July 2020 and October 2021. The study was approved by the Ethics Committee of the Bavarian Medical Association (No. 19096), registered at clinicalTrials.gov (NCT051826745), and conducted according to the Declaration of Helsinki.

The day-clinic program used in this study includes ten weekly sessions of 6 h each, for a total of 60 h, and is adapted to the needs of CD patients. In the sessions with the main focus on mindfulness, patients learn about mind-body medicine using meditative movements (e.g., yoga, tai chi, qigong), relaxation techniques (e.g., meditation, body scan, breathing relaxation), endurance training (e.g., fast walking/Nordic walking, swimming, cycling), self-care strategies, stress management, nutritional counselling with focus on a plant-based Mediterranean diet, naturopathy self-help strategies and herbal remedies. During day-clinic, all patients must write a diary regarding their stress coping activities (amount of conducted relaxation techniques, meditative movements, and endurance training). At baseline and after the day-clinic program, patients’ perceived stress levels were measured. In week 24, qualitative interviews were conducted with the intervention group regarding implementation of strategies learned during their day-clinic visits in their daily lives. Participants in the control group received a single 90-min educational session on mind-body medicine and complementary self-care strategies, either on site or virtually, approximately two weeks following randomization.

**Fig. 1:**
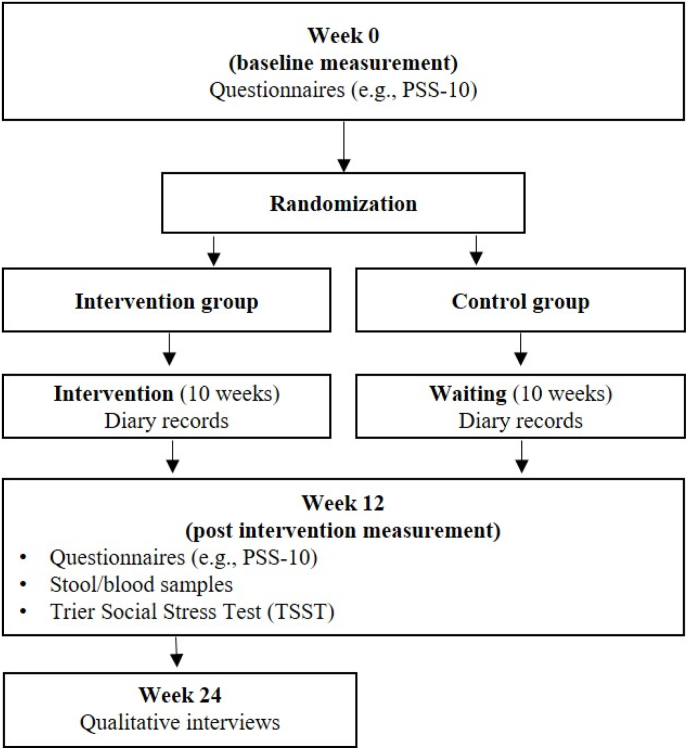
Overall study design.

Results of a sub-study applying a laboratory stress protocol (TSST) (Kirschbaum et al., 1993) as part of this RCT are reported. The overall study design is depicted in Fig. 1.

### 2.2 Participants

The RCT (Bauer et al., 2024) included 42 patients (f, m) between 18 and 75 years of age with a confirmed diagnosis of Crohn's disease by a specialist based on medical history, endoscopy and histology. Five patients dropped-out, resulting in 37 patients receiving either intervention (n = 19) or not (n = 18). Inclusion criterion were stable medication for at least three months, regardless of whether glucocorticoids, immunosuppressants, azathioprine, or other drug treatments are taken according to the guideline. Lastly, a signed informed consent form must be available. Exclusion criteria for study participation were an infectious or chronically active CD, a complete colectomy, severe mental illness (e.g., major depression requiring treatment, addiction, schizophrenia) or severe comorbid somatic diseases (e.g., diabetes mellitus, oncological disease). Women during pregnancy and patients enrolled in a stress reduction program or clinical trials of psychological interventions were also unable to participate in the study.

A power analysis for our sub-study indicated that the minimum sample size to yield a statistical power of at least .8 with α = .05 and a small-to-medium effect size (d = 0.25) is 34. In our sub-study, 33 patients (23 female, 10 male) with CD participated in the TSST of which 19 (13 female, 6 male; age: *M* = 49.6; *SD* = 13.1) were part of the intervention group and 14 (10 female, 4 male; age: *M* = 45.6; *SD* = 11.6) of the control group in the RCT. Medication at week 12 was not different between the intervention and control group regarding CD targeted biologicals [χ^2^ (1, *N* = 32) = 0.05, *p* = .821], immunosuppressive drugs [χ^2^ (1, *N* = 32) = 0.80, *p* = .370], glucocorticoids [χ^2^ (1, *N* = 32) = 0.37, *p* = .542]).

The qualitative interview sample consisted of all patients (n = 19) from the intervention group.

### 2.3 Procedure and measures

*Perceived Stress Scale (PSS-10)* (Klein et al., 2016). The German version of the Perceived Stress Scale was used to assess the individuals’ perceived stress level at baseline and week 12 (same time frame as TSST). It is administered as a 10-question survey, assessing how unpredictable, uncontrollable, and overwhelming respondents have found their lives over the past 14 days.The total score ranges from 0 to 40 (rating scale from 0 to 4).

*Diary records.* During day-clinic, each participant was provided with a diary to record their stress coping activities. The diary was structured to include sections for different types of activities, such as relaxation techniques, meditative movements, and endurance training. Relaxation techniques include practices like meditation, deep breathing exercises, progressive muscle relaxation or body scan. Meditative movements focus on activities that combine movement with mindfulness, such as yoga, tai chi, or qigong. Endurance training comprises physical activities aimed at building endurance and resilience. This could involve activities like jogging, swimming, cycling, or walking (fast walking/Nordic walking). The entries allow tracking the duration (in minutes) of each session and the progress of activity during the course of the study.

*Trier Social Stress Test (TSST)* (Kirschbaum et al., 1993). In the first part, participants are asked to put themselves in the role of a job applicant and to explain in a 5-min free speech to a selection committee consisting of two psychologist (actors in doctor's coats) which personal characteristics make them suitable for this field of work. Participants are informed that the observers will also focus on their nonverbal behavior and that a video camera and a tape recorder will record their speech. To prepare for their speech, the subjects are given 5 min to take notes, which may however not be used during the speech. The selection committee will remain as neutral as possible throughout the participants' speech and, if necessary, will indicate in a standardized manner any time remaining if the participants speak for less than 5 min. After this simulated job interview is finished, a second challenge follows for the participants: they are asked to count down from the number 1022 in increments of 13 as correctly and as quickly as possible. Each time they make a mistake, participants have to start again from scratch. Participants will be interrupted after 5 min and an experimenter informs the participants about the aim and purpose of the study and assures them that neither the video nor the tape recording of the speech given will be analyzed (Kirschbaum et al., 1993).

**Fig. 2:**
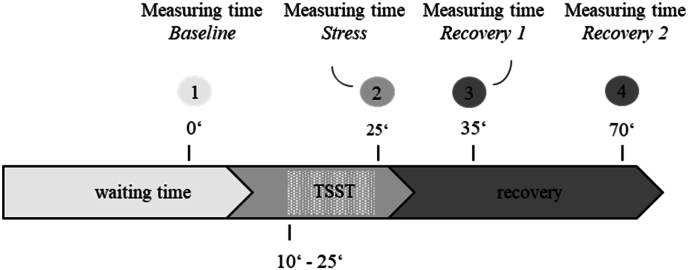
Procedure of the Trier Social Stress Test (TSST) including the four measuring time points in minutes (Baseline, Stress, Recovery 1, Recovery 2).

Besides vital signs such as blood pressure and pulse, at four timepoints of TSST (Baseline/minute 0, Stress/minute 25, Recovery 1/minute 35, Recovery 2/minute 70, see Fig. 2), blood samples were taken to determine immune and endocrine parameters. WBC differential for leucocyte and platelet counts was analyzed by the local clinical laboratory. For cortisol, venous blood was collected in tubes containing EDTA (S-Monovette, Sarstedt, Nümbrecht, Germany) and plasma was separated by centrifugation (2000g, 10min, 4 °C) and stored at −80 °C until analyses.

*Qualitative interviews*. Nineteen semi-structured interviews (n = 19) were conducted in week 24 using an interview guideline that, in addition to fixed interview topics, allows for unexpected insights and allows participants to contribute their own topics and opinions (Saldana, 2011). This approach can be grounded in the field of symbolic interactionism and phenomenology, as the interest in knowledge lies in the subjective views and life circumstances of the patients, and the data were obtained from communication (Flick et al., 2004). The interviews, which lasted an average of 40 min, were audio-recorded, transcribed verbatim, and anonymized. Reflexive thematic analysis was applied to identify relevant themes and patterns in the participants' subjective meanings (Braun and Clarke, 2006, 2022). The interdisciplinary research team used MAXQDA software for the coding processes. The analysis procedure and results were discussed and reflected upon by the research team.

## 3 Results

Repeated measures ANOVAs were calculated for all dependent variables of interest with the between-subjects factor group (intervention, control) and within-subject factor measuring point (PSS-10: 1, 2; Diary records: 0–12; TSST: 1, 2, 3, 4). Participants with missing values were excluded from analysis for the respective dependent variable.

*Perceived Stress (PSS-10)*. One participant had to be excluded from analysis because of inconsistent response patterns to validity items. There was a significant main effect of measuring point, *F* (1,30) = 11.58, *p* = .002, η_p_^2^ = .279, showing overall lower perceived stress at week 12 compared to baseline and no effect of group, *F* (1,30) = 0.005, *p* = .947, η_p_^2^ < .001. There was a significant interaction between group and measuring point, *F* (1,30) = 4.92, *p* = .034, η_p_^2^ = .141, revealing at week 12 (*M* = 14.2; *SD* = 6.5) a significant reduction in perceived stress in the intervention group compared to baseline (*M* = 20.6; *SD* = 7.1; *p* < .001), whereas there was no change in perceived stress in the control group (baseline: *M* = 17.9; *SD* = 7.2; week 12: *M* = 16.6; *SD* = 5.5; *p* = .436).

**Fig. 3:**
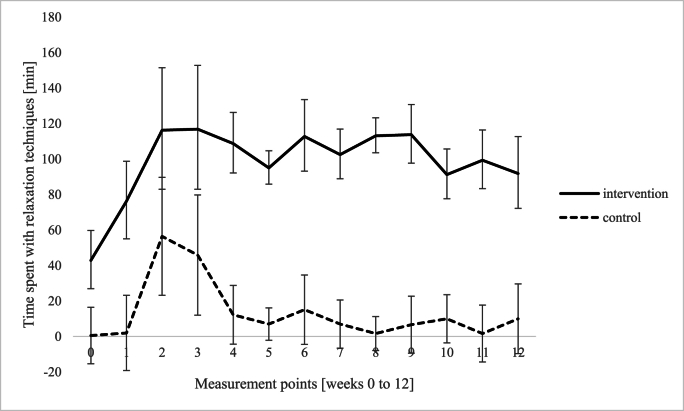
Differences between intervention and control group with regard to relaxation techniques (e.g. meditation, body scan, breathing relaxation).

**Fig. 4:**
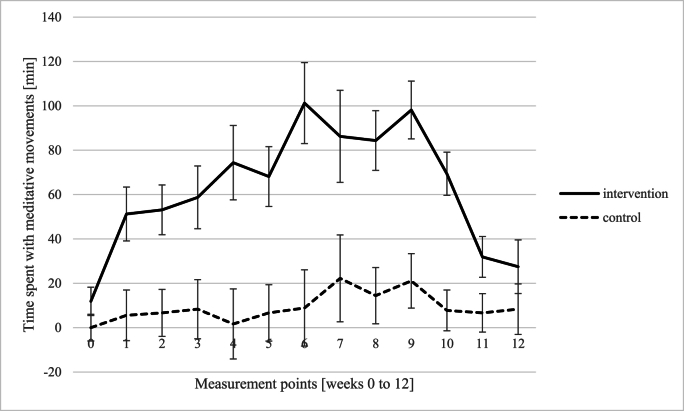
Differences between intervention and control group with regard to meditative movements (e.g., yoga, qigong, tai chi).

**Fig. 5:**
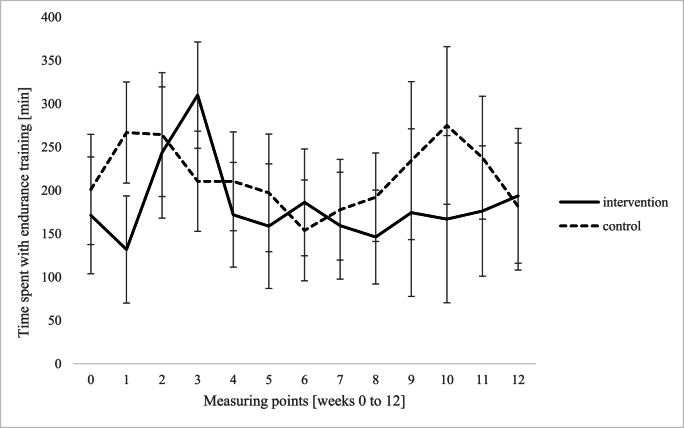
No differences between intervention and control group with regard to endurance training (e.g. fast/Nordic walking, swimming, cycling).

*Diary records.* Diary records (12 weeks) of relaxation techniques demonstrated a significant main effect of group, *F* (1,15) = 18.43, *p* < .001, η_p_^2^ = .551, i.e., a significant higher amount of relaxation techniques among the intervention group compared to the control group. Furthermore, there was a significant main effect of measuring point, *F* (12,180) = 2.29, *p* = .010, η_p_^2^ = .132, and no interaction between measuring point and group, *F* (12, 180) = 0.81, *p* = .645, η_p_^2^ = .051 (see Fig. 3). Regarding meditative movements, there was a significant main effect of group, *F* (1,15) = 23.85, *p* < .001, η_p_^2^ = .614, showing a significantly higher amount of meditative movements in the intervention group compared to the control group. There was a significant main effect of measuring point, *F* (12, 180) = 4.09, *p* < .001, η_p_^2^ = .214, and a significant interaction between measuring point and group, *F* (12,180) = 2.26, *p* = .011, η_p_^2^ = .131, revealing significant differences between the groups from week 1 until week 11 (see Fig. 4). With regard to endurance training, there was no effect of group, *F* (1,15) = .14, *p* = .712, η_p_^2^ = .009, measuring point, *F* (12,180) = 1.23, *p* = .263, η_p_^2^ = .076, and the factors did not interact, *F* (12,180) = 1.20, *p* = .286, η_p_^2^ = .074 (see Fig. 5).

Additionally, mediation analyses (using PROCESS v4.2 by Hayes, A.F.) were done to evaluate potentially mediating effects of meditative movements and relaxation techniques on perceived stress (PSS-10 at week 12). The first mediation analysis revealed that participation in our program significantly predicts amount of time spent with meditative movements, *R*^*2*^ = .434, *F* (1,24) = 17.01, *p* < .001, indicating that 43.4 % of the variance in meditative movements is explained by participation in our program. The coefficient indicated that participation in our program had a significant positive direct effect on meditative movements (*B* = 83.95, *SE* = 20.36, *t* (25) = 4.12, *p* < .001). In the combined model predicting perceived stress level, both participation in the program and meditative movements were included as predictors, resulting in a non-significant model, *R*^*2*^ = .021, *F* (2,23) = .88, *p* = .429, i.e. participation in our program and meditative movements together do not have a significant explanatory contribution regarding perceived stress. The second mediation analysis revealed that participation in our program significantly predicts amount of time spent with relaxation techniques, *R*^*2*^ = .508, *F* (1,24) = 22.87, *p* < .001, indicating that 50.8 % of the variance in relaxation techniques is explained by participation in our program. The coefficient indicated that participation in our program had a significant positive direct effect on relaxation techniques (*B* = 48.587, *SE* = 10.16, *t* (25) = 4.78, *p* < .001). In the combined model predicting perceived stress level, both participation in the program and relaxation techniques were included as predictors, resulting in a non-significant model, *R*^*2*^ = .042, *F* (2,23) = .48, *p* = .623, i.e. participation in our program and relaxation techniques together do not have a significant explanatory contribution regarding perceived stress.

### 3.1 Trier social stress test

*Vital signs.* Regarding pulse, there was a significant main effect of measuring point, *F* (3,87) = 4.00, *p* = .010, η_p_^2^ = .121, with an increase in pulse from baseline to the stress time point for both groups (*p* = .015), but no difference between groups, *F* (1,29) = .25, *p* = .625, η_p_^2^ = .008, and no interaction between group and measuring point, *F* (3,87) = .62, *p* = .604, η_p_^2^ = .021. Regarding blood pressure, for the systolic value, there was a significant main effect of measuring point, *F* (3,87) = 200.14, *p* < .001, η_p_^2^ = .873, with a significant drop at recovery 2 compared to baseline, stress and to recovery 1 for both groups (all *p*s < .001), but no difference between groups, *F* (1,29) = 3.13, *p* = .087, η_p_^2^ = .097, and no interaction between group and measuring point, *F* (3,87) = 1.86, *p* = .143, η_p_^2^ = .060. For the diastolic value, there was a significant main effect of measuring point, *F* (3,84) = 10.28, *p* < .001, η_p_^2^ = .268, with an increase from baseline to the stress time point and recovery 1 and a significant drop from the stress time point to recovery 2 for both groups (all *p*s < .030), but no difference between groups, *F* (1,28) = .12, *p* = .736, η_p_^2^ = .004, and no interaction between group and measuring point, *F* (3,84) = .17, *p* = .918, η_p_^2^ = .006.

**Fig. 6:**
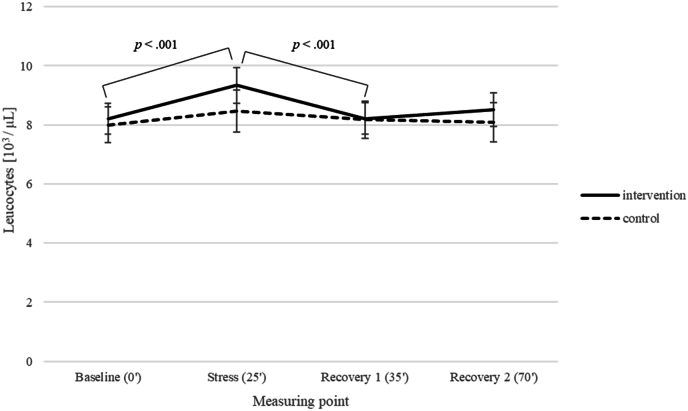
Leucocyte count of intervention and control group with regard to the four measuring points of the TSST.

**Fig. 7:**
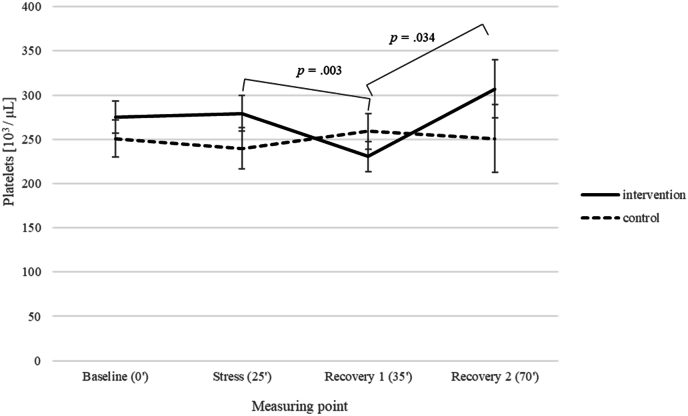
Platelet count of intervention and control group with regard to the four measuring points of the TSST.

**Fig. 8:**
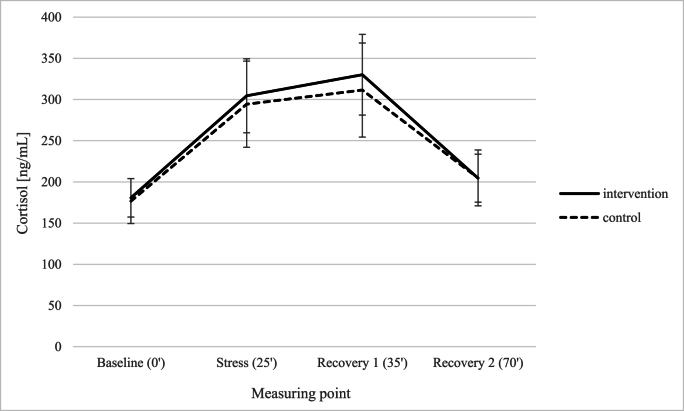
Cortisol levels of intervention and control group with regard to the four measuring points of the TSST.

*Immune and endocrine parameters.* Blood samples taken during acute stress in the TSST at week 12 showed for leucocytes no effect of group, *F* (1,31) = .21, *p* = .647, η_p_^2^ = .007, a significant main effect of measuring point, *F* (3,93) = 10.53, *p* < .001, η_p_^2^ = .076, and a marginally significant interaction of time point and group, *F* (3,93) = 2.56, *p* = .060, η_p_^2^ = .076 (medium effect size). The Bayes factor upper bound (BFB; using the formula provided by Held and Ott, 2018) (Held and Ott, 2018)) for a p-value of .060 results in a BFB of 2.18, i.e. the alternative hypothesis is more than twice as likely as the null hypothesis. The interaction revealed a significant increase of leucocytes in the intervention group at the stress time point in comparison to baseline, followed by a significant drop in leucocytes at recovery 1 and 2 (all ps < .02), but no change in leucocytes in the control group (all ps > .500) (see Fig. 6). For platelets, there was no effect of group, *F* (1,31) = .61, *p* = .442, η_p_^2^ = .019, no effect of measuring point, *F* (3,93) = 1.54, *p* = .208, η_p_^2^ = .047, and a marginally significant interaction for measuring point and group, *F* (3,93) = 2.64, *p* = .054, η_p_^2^ = .079 (medium effect size). The BFB (Held and Ott, 2018) for a p-value of .054 equals 2.33, i.e. the alternative hypothesis is again more than twice as likely as the null hypothesis, revealing a significant drop in platelets level at recovery 1 compared to baseline (*p* = .021) and stress (*p* = .003), followed by a significant increase in platelets at recovery 2 compared to recovery 1 (*p* = .034) in the intervention group. Again, there was no change in platelets for all measuring points for the control group (all *p*s = 1.000) (see Fig. 7). Regarding cortisol, there was a significant main effect of measuring point, *F* (3,93) = 34.06, *p* < .001, η_p_^2^ = .523, showing a significant increase of cortisol from baseline to the stress time point and recovery 1 (all *p*s < .001) followed by a significant drop of cortisol for recovery 2 compared to the stress time point and recovery 1 (all *p*s < .001), no effect of group, *F* (1,31) = .02, *p* = .882, η_p_^2^ = .001, and no interaction for measuring point and group, *F* (3,93) = .12, *p* = .949, η_p_^2^ = .004 (see Fig. 8).

*Qualitative interviews*. The analyses of the narratives reveal a pattern indicating a *perceived reduction in stress in daily life*. Participants who participated in the multimodal lifestyle modification and stress reduction program reported a generally higher level of calmness and feel less stress, which from their perspective was previously more pronounced. This is apparent in particularly stressful situations. Individuals reported that they *no longer experience stress as quickly* as before the intervention and *have learned effective ways to manage stress* when it does occur. They feel that they have learned appropriate strategies to better cope with stressful situations:

“Well, at work. I no longer feel the stress I used to. I used to be much more likely to fly off the handle or be affected by the stress of work, which I no longer feel at all”. (P08, female, 56 years)

“[…] that I deal with stress differently, that has already changed. Somehow, I've learned to deal with stress in different way.” (P08, female, 56 years)

On the one hand, the *perception of the stress situation has been sensitized*, which now gives patients the opportunity to react and actively intervene in stressful situations:

“I consciously recognize stressful situations that I may not have noticed before. My entire perspective on stress is also different now and, accordingly, the way I deal with it, is also different. I am now consciously aware of it and, of course, when I realize it that I am now […] also trying to take countermeasures, such as doing sport again in the evening or perhaps a relaxation exercise or something similar to bring my body back into balance. That means my perception and the way I deal with stress is completely different. And I now have a nice package of tools and know how I can counteract this accordingly.” (P10, male, 28 years)

“When I have the feeling that something is stressing me out. Now I have to put up a stop sign for myself. Then I also have a few [new learned] instructions.” (P07, female, 64 years)

On the other hand, *specific techniques* could be applied. In almost all interviews, stress management techniques were reported to be used in daily life. It is obvious that short, stress-reducing units are used to improve, especially acute stress situations. These exercises are flexible in terms of location and time, yet effective from the patient's perspective. They are often combined with home exercises:

“But I try to do the things I've learnt, such as breathing techniques, when I notice that I'm stressed or get stressed, I try to do breathing exercises or progressive muscle relaxation. Or autogenic training, so that I can calm down again, so that I become calmer [ …], since I took part in the study, it became better, I don't let myself get so stressed anymore.” (P14, female, 53 years)

“When I simply notice that I'm not feeling well at all, then I do these breathing minis exercises, that also works very quickly […] that also works in between.” (P06, female, 37 years)

“Qigong, or the little short breaks, these breathing exercises […] And I did them during day-clinic or afterwards whenever things got exhausting or stressful or when I felt stressed, then I simply used these things to calm myself down. […] And that was quite good.” (P01, male, 61 years)

The content of the TSST (Kirschbaum et al., 1993) was not the subject of the qualitative study and therefore not the subject of the interviews, but some interviewees talked about the test and highlighted the perception of a real stress situation:

“And what was horrible for me was that test day. […] This stress test. I thought it was extremely hard, but also brilliantly good. I thought, what's going on? It was done professionally. It couldn't have been better. […] And I thought to myself, these people are either such narcissists or they were professional actors.” (P05, male, 52 years)

## 4 Discussion

The current study underscores the effectiveness of a 10-week comprehensive stress management and lifestyle modification (LSM) program in positively influencing subjective stress perception and possibly also the objective immune response among patients diagnosed with CD. The LSM program led to a remarkable reduction in stress by applying mindfulness-based stress reduction techniques in everyday life, and perhaps to changes in sensitivity of leucocytes and platelets in acute stress.

Our post intervention analysis at week 12 revealed a *significant reduction in perceived stress in the intervention group* compared to their initial baseline measurements. In contrast, no such reduction was observed in the control group over the same period of time. In addition, there were clear reports in the *qualitative interviews* of the IG of a perceived reduction in stress in everyday life and that the patients no longer felt stressed so quickly as before the intervention. It has been proven that lifestyle interventions can have a positive effect on the perception of stress in patients with IBD (Elsenbruch et al., 2005; Langhorst et al., 2020; Schlee et al., 2022). Furthermore, our finding is in line with the results of Koch et al. (2021) in ulcerative colitis. There, patients’ perceived stress declined significantly after participation in the LSM program and even lasted until week 24. The reported decrease in perceived stress in this study might be the result of a reduction in chronic stress through our day-clinic program. But what did specifically contribute to this observation: an individual measure or the program as a whole, with its different focuses in various areas? Mediation analysis has shown that no measure alone, for example “meditative movements” or “relaxation”, can reduce stress to this extent. In addition, only the amount of time spent was recorded in the diaries. It can be assumed that there were also qualitative differences in implementation due to the instructions in the program as opposed to the short training course (CG). The changes in perceived stress were brought about by the guided overall program with a variety of self-care strategies.

*Diary records* in our study display a significantly higher amount of relaxation techniques among the IG compared to the CG. Additionally, participants in the IG demonstrated a notable uptake in meditative movements like yoga, qigong or tai chi, with significant interaction between time and group. The *interviews* revealed a broad use of different stress management techniques, but with a clear focus on short units that can be applied flexibly in everyday life, such as breathing and relaxation techniques. Nearly all patients reported that they were able to incorporate stress management techniques into their daily routines, individually and as required. This improved self-awareness and the ability to deal with stress. By participating in an LSM program, participants have acquired effective strategies for managing stress (Schlee et al.) and consistently report a heightened sense of calmness in their daily lives.

As it is known, techniques like relaxation, yoga and mindfulness can have positive impact on stress regulation in patients with IBD (Cramer et al., 2017; Koch et al., 2020; Mizrahi et al., 2012). However, there were no discernible differences between the groups regarding the measure of endurance training. The control group did just as much exercise as the IG, but did not benefit as much regarding stress perception. Obviously, the extensive utilization of additional self-care techniques seemed to make the crucial difference in (chronic) stress reduction between the groups. Techniques such as meditative movements and relaxation, quantifiable here through the diary, as well as the entire modules of the LSM program influence the perception of stress as such. The reason could be that interventions that comprise acceptance and mindfulness procedures can increase psychological flexibility and reduce stress (Wersebe et al., 2018). In ulcerative colitis, Schlee et al. (2022) described that the strong focus on personal responsibility and self-care was vital for reducing psychological stress and physical symptoms. As a result, both the patient's well-being and quality of life improved. In our main study (Bauer et al., 2024; Schlee et al.) on CD, equivalent analyses yielded similar impact of stress management in psychological, physical, and social domains (such as improvements of quality of life and relevant fecal markers). The role of exercise, such as endurance training, as a method for stress prevention is not yet fully understood and warrants further investigation (Hashash and Binion, 2017).

Our results regarding perceived stress (significant interaction) and stress reduction techniques (from diaries; clear main effects of group), indicate, that the changes in our intervention group across time are caused by our intervention.

It is by now widely recognized that acute and chronic stress have an impact on the immune system. Acute stress may have a stimulating effect on the immune system, while in the case of chronic stress the immune system may be down-regulated. However, there is considerable individual variability in the immune response to stress. This seems to a large extent to be determined by the subject's way of dealing with stress (Olff, 1999). In the present study, we could successfully induce acute psychological stress by TSST as reported by participants and seen at the cortisol reaction. Furthermore, there were hints to an altered immune response to acute stress in leucocytes and platelets in the intervention group in comparison to control group. Acute stress led to a significant increase of leucocytes in the intervention group compared to baseline. Human studies have consistently indicated that acute stress elevates blood immune cell counts compared to resting states (e.g. Schakel et al., 2019; Mills et al., 1995). This increase in leucocyte mobilization is part of the "fight-or-flight" response, where the body's stress hormones, such as adrenaline and cortisol, stimulate the rapid production and release of white blood cells from bone marrow and other reservoirs into the bloodstream. The immune system prepares itself for possible injuries and infections or both. Assessing the immune system's response to activating stimuli provides a reliable measure of immunocompetence, serving as a biologically valid marker of an effective immune response (Schakel et al., 2019). The drop of leucocyte levels in the recovery phase of the TSST might represent leucocyte traffic out of the blood and into tissues (Dhabhar et al., 2012). Stress-induced leucocyte redistribution may be a crucial survival mechanism that directs leucocyte subpopulations to specific target organs during stress, significantly enhancing the speed, efficacy, and regulation of the immune response (Dhabhar et al., 2012).

The observation that an increase in leucocytes as a reaction to acute stress could only be triggered in the IG, seems to indicate that the participants are able to form efficient immune responses as a result of the LSM program. By reducing chronic stress, as shown by the decrease in perceived stress levels, responses to acute stress could possibly be modified. It is known that the immune responses of chronically stressed people to acute stress differ from those of healthy people, suggesting a disorder in the sympathetic, neuroendocrine, and immunological mediated processes (Pike et al., 1997). Impairments in the regulation of those processes may directly affect, among others, receptor density and sensitivity of immune cells, their stimulability and mobility, as seen on the percentage of circulating immune cells (Dragoş and Tănăsescu, 2010). In contrast to acute stress, chronic stress impairs T cell function (Dragoş and Tănăsescu, 2010). All results of our sub-study referring to the efficacy of immune response, are supported by the findings regarding immunological regulation examined as part of another sub-study by Mekes-Adamczyk et al. (Mekes-Adamczyk et al., 2025) of the 10.13039/100014144RCT (Bauer et al., 2024). Mekes-Adamczyk et al. (Mekes-Adamczyk et al., 2025) compared circulating CD4^+^ T cells from CD patients (same intervention group as in the present study) with the cells of healthy blood donors in the course of the study. They observed reduced T cell frequencies in the blood of the patients and a significant correlation between the quality of life improvement and those cell frequencies. They concluded that the LSM program can potentially restore the CD4^+^ T cell profile of CD patients to levels comparable to those of healthy blood donors (Mekes-Adamczyk et al., 2025). Despite substantial heterogeneity across studies, a review by Black and Slavich (2016) with a specific focus on circulating markers of inflammation and number of immune cells, found evidence that mindfulness meditation can improve immune system dynamics. In their 2019 meta-analytic review, Schakel et al. (2019) found that psychological interventions, such as stress management, cognitive-behavioral therapy, and mindfulness practices, yielded a small-to-medium effect size in enhancing immune function. This effect was consistent across multiple studies, indicating that these interventions can contribute to better immune responses, though the magnitude of the improvement varies. Therefore, our results seem to be in line with the latest findings in this area of research.

Platelets represent the major cell type involved in the regulation of hemostasis and thrombosis. In addition to performing hemostatic roles, platelets can among others interact with the vascular endothelium and leucocytes and are therefore important coordinators of inflammation (Thomas and Storey, 2015), and can influence both innate and adaptive immune responses. Laboratory stress is associated with the activation of blood platelets (Koudouovoh-Tripp et al., 2021; Mundal, 1996; Levine et al., 1985) reflected by changes in platelet bioactivity and aggregation (Bentur et al., 2018). Platelets activated in the circulation engage in a crosstalk with endothelial cells to promote the local immune response and limit pathogen invasion through immunothrombosis (Coenen et al., 2017). Activated platelets physically interact with endothelial cells (Coenen et al., 2017) and immune cells. Mental stress has been shown to increase platelet aggregability (von Känel et al., 2001; Naesh et al., 1993). In order to clarify further stress-induced platelet activation with special reference to the period after the stress, healthy volunteers were studied during and for 1 h after a mental stress test (Naesh et al., 1993). Platelet aggregability was unaffected during the test but decreased following the stress. The researchers concluded that platelets are activated during mental stress and that this activation involves a post-stress release of vasoactive compounds from platelets (Naesh et al., 1993). In our study, acute stress led to a significant drop in platelet levels after stress exposure, followed by a significant increase, both in the intervention group. These observations seem also to be part of the "fight-or-flight" response. Fighting and fleeing carries the risk of injury, which can allow infectious agents to enter the bloodstream or penetrate the skin. Any skin wound is likely to harbor pathogens that could proliferate and lead to infection (Williams and Leaper, 1998). Stress-induced changes in the immune system that enhance wound repair and prevent infections would be adaptive, likely selected alongside other physiological changes that increased evolutionary fitness (Segerstrom and Miller, 2004). Acute stress upregulates parameters of natural immunity, the branch of the immune system in which most changes occur, which requires only minimal time and energy investment to defend against invaders (Segerstrom and Miller, 2004). Schakel et al. (2019) highlighted that the most compelling evidence for optimizing immune function through psychological interventions was observed in cases of wound healing (medium effect size). Faster wound healing indicates a more effective immune response. As stressors became more chronic, the potential adaptiveness of the immune changes decreased (Segerstrom and Miller, 2004). Research gives hint that the recovery of platelet function following an acute mental stress exposure is significantly impaired by chronic stress (Koudouovoh-Tripp et al., 2021). Of note is, that aggregation is affected by chronic and acute mental stress. In healthy individuals, platelet-leucocyte aggregation (PLA) returns to baseline levels within 20–45 min following an acute stress session. In contrast, PLA gradually increases up to 75 min post-stress in cardiovascular disease (CVD) patients (Strike et al., 2004), suggesting that CVD patients experience prolonged platelet activation under stress (Sandrini et al., 2020). The observed drop in platelet count in our intervention group after stress exposure in our sub-study could be explained by stress-induced activation and aggregation. The observed increase in the recovery phase could be interpreted as the rapid restoration of the basal state.

Acute stress triggers the “fight-or-flight” response with activation of the HPA (hypothalamic-pituitary-adrenal) axis and the release of the stress hormone *cortisol* (Rohleder, 2012). Interestingly, no physiological differences in response to acute stress were found between the groups in our sub-study. *Pulse and blood pressure* were not considerably different. Participants in both groups did not differ significantly in cortisol levels during the TSST. In their study on ulcerative colitis, Koch et al. (2021) observed group differences in pulse rates, but also found no significant differences in cortisol levels. In the study of Rosenkranz et al. (2013) with healthy individuals, participants who received a stress-reducing mindfulness intervention showed a lower post-stress (i.e., after the TSST) inflammatory response compared to a control group, despite equivalent stress-evoked cortisol levels. So, even if the cortisol levels in our sub-study are similarly high in both groups, it can still be assumed that the regulation of immune cells may differ. Chronic stress is related to a decrease in the glucocorticoid sensitivity of peripheral immune cells (Rohleder, 2012). By reducing perceived chronic stress in our intervention group, the sensitivity could be restored.

### 4.1 Strength and limitations

One can resume that our findings might indicate that an LSM program can reduce or eliminate disturbed functioning of the stress adaptation systems in CD. Our marginal significant results might be due to the fact that we did not completely achieve the required sample size. However, the Bayesian Factor and effect sizes argue at least weakly in favor of our interpretation. Therefore, our conclusions are not definitive and require further validation in larger studies.

It is also possible that some differences between the groups were too subtle to be detected. Due to the lack of group differences in immune function, the within-group differences are not sufficient to be able to attribute the effects completely to the intervention. The effects should therefore be regarded as preliminary and further studies are needed to confirm these results. To obtain more precise information about the changes in immune response, it would be useful to take a closer look at the inflammation, such as through the intestinal profile. There is no doubt that stress has a negative impact on CD and effective interventions to reduce stress are needed.

## 5 Conclusions

The findings suggest that a day-clinic LSM program can be beneficial for CD patients by improving health-related quality of life and relevant fecal markers, reducing perceived stress and possibly enhancing immune response to acute psychological stress. These improvements highlight the potential of integrating multimodal stress management and lifestyle changes into CD treatment towards a holistic care. Further research is needed to explore the long-term effects and underlying mechanisms of these changes.

## CRediT authorship contribution statement

**Özlem Öznur:** Writing – review & editing, Writing – original draft, Methodology, Conceptualization. **Sandra Utz:** Writing – review & editing, Writing – original draft, Methodology, Formal analysis. **Christoph Schlee:** Writing – review & editing, Writing – original draft, Methodology, Formal analysis, Conceptualization. **Jost Langhorst:** Writing – review & editing, Supervision, Resources, Project administration, Funding acquisition, Conceptualization.

## Funding sources

This study was supported by a grant of the Bavarian State Ministry for Health and Care (Germany), which was administrated through the funding program Gesund.Leben.Bayern (in English: Healthy.Living.Bavaria; reference number: GE7-2497-GLB-19-V4).

## Declaration of competing interest

The authors declare that they have no known competing financial interests or personal relationships that could have appeared to influence the work reported in this paper.

## Data Availability

Data will be made available on request.
